# Engineering of Chinese Hamster Ovary Cells With NDPK-A to Enhance DNA Nuclear Delivery Combined With EBNA1 Plasmid Maintenance Gives Improved Exogenous Transient Reporter, mAb and SARS-CoV-2 Spike Protein Expression

**DOI:** 10.3389/fbioe.2021.679448

**Published:** 2021-06-04

**Authors:** James D. Budge, Robert J. Young, Christopher Mark Smales

**Affiliations:** ^1^Industrial Biotechnology Centre, School of Biosciences, University of Kent, Canterbury, United Kingdom; ^2^R&D Cell Engineering Group, Lonza Biologics, Chesterford Research Park, Saffron Walden, United Kingdom

**Keywords:** transient gene expression, nuclear localisation signal, nucleoside diphosphate kinase, Chinese hamster ovary cells, Epstein Barr Nuclear Antigen-1, SARS-CoV-2 spike protein

## Abstract

Transient gene expression (TGE) in mammalian cells is a method of rapidly generating recombinant protein material for initial characterisation studies that does not require time-consuming processes associated with stable cell line construction. High TGE yields are heavily dependent on efficient delivery of plasmid DNA across both the plasma and nuclear membranes. Here, we harness the protein nucleoside diphosphate kinase (NDPK-A) that contains a nuclear localisation signal (NLS) to enhance DNA delivery into the nucleus of CHO cells. We show that co-expression of NDPK-A during transient expression results in improved transfection efficiency in CHO cells, presumably due to enhanced transportation of plasmid DNA into the nucleus via the nuclear pore complex. Furthermore, introduction of the Epstein Barr Nuclear Antigen-1 (EBNA-1), a protein that is capable of inducing extrachromosomal maintenance, when coupled with complementary *oriP* elements on a transient plasmid, was utilised to reduce the effect of plasmid dilution. Whilst there was attenuated growth upon introduction of the EBNA-1 system into CHO cells, when both NDPK-A nuclear import and EBNA-1 mediated technologies were employed together this resulted in enhanced transient recombinant protein yields superior to those generated using either approach independently, including when expressing the complex SARS-CoV-2 spike (S) glycoprotein.

## Introduction

Chinese hamster ovary (CHO) cells are the most commonly utilised expression hosts for the production of recombinant biotherapeutic proteins such as monoclonal antibodies (mAbs) as they are able to generate complex, multi-domain, multi-chain proteins with human-like post-translational modifications (Godfrey et al., 2017). Furthermore, the number of commercial recombinant products in the clinic has consistently increased in recent years largely due to an increase in the number of mAb approvals but also as a result of the emergence of novel format molecules such as Fc-fusion proteins and bispecific antibodies (Walsh, 2018). Technologies and processes underpinning the generation of recombinant material in CHO cells have been extensively developed enabling manufacturers to achieve product titres greater than 5 g/L of mAb (Marichal-Gallardo and Álvarez, 2012; Budge et al., 2019). Despite these considerable advances, the processes required to stably integrate genes of interest into a CHO host cell chromosome followed by screening, selection and adaption of a high yielding clone which is stable in both product quality and productivity of a desired molecule takes up to 12 months (Wurm, 2004). The generation of stable clonal cell lines is time consuming and not always required during early drug discovery processes where it may be necessary to rapidly assess multiple potential therapeutic targets. Instead, transient gene expression (TGE) is often the favoured production platform in this scenario since it is possible to generate material for initial characterisation studies in as little as 1–2 weeks. It is also preferable to employ the same production host to transiently generate therapeutic candidates as will be used to stably generate the final product. For example, this can give greater confidence of obtaining similar post-translational modifications and product properties in transiently generated material as in stably generated material to ensure that candidates evaluated early in the drug discovery process are indicative of the final clinical molecule (Povey et al., 2014).

In comparison to stable production, recombinant titres from TGE are typically compromised in favour of speed and there are a number of limitations that are specific to transient platforms; not least of which is the requirement for efficient DNA delivery into the nucleus of the host. Migration of plasmid DNA (pDNA) to the nucleus without being degraded is vital to produce recombinant material since it is the site of transcription of the recombinant DNA to generate mRNA. Although many chemical and physical transfection methods have been developed to transfer DNA across the cell membrane into the cytoplasm, the process by which exogenous DNA subsequently reaches the nucleus is not well defined. Entry of pDNA into the nucleus has been shown to be maximal in proliferating cells and is thought to be aided by the disassembly of the nuclear envelope during mitosis which removes the nuclear membrane barrier (Dean et al., 2005). Alternatively, nuclear import of pDNA can be facilitated by a signal mediated pathway through the nuclear pore complex (NPC) in non-dividing cells, as is conventional for macromolecular exchange between the cytoplasm and the nucleus (Gasiorowski and Dean, 2003). Delivery of molecules from the cytoplasm to the nucleus through the NPC is facilitated by binding of importin which, in turn, delivers cargo in a Ran/GTP dependent mechanism (Pemberton and Paschal, 2005). The rate of import of pDNA through the NPC is largely dependent on plasmid size and structure, i.e., supercoiled pDNA more readily traverses the nuclear membrane than linear DNA. It has been shown that only 0.1% of naked DNA or 1% of polymer attached DNA migrates into the nucleus following microinjection into the cytoplasm in COS7 cells (Pollard et al., 1998).

Enhancement of nuclear import of transfected pDNA has been reported through two major chaperone mediated approaches. Firstly, proteins which bind to pDNA and contain nuclear localisation signals (NLSs) have been employed and form a complex with importin facilitating the migration of pDNA into the nucleus through the nuclear pore complex (NPC) (Subramanian et al., 1999; Zanta et al., 1999; Gourbatsi et al., 2006). Similarly, the inclusion of specific nucleotide sequences, known as DNA nuclear target sequences (DTSs), in pDNA can bind to proteins that bind DTSs and contain endogenous NLSs, facilitating nuclear import through the NPC (Dean, 1997). Nucleoside diphosphokinase A (NDPK-A) and Visual Homeobox 2 (Chx10) transcription factors have been identified as cytoplasmic NLS containing proteins capable of chaperoning pDNA in non-dividing HeLa cells (Munkonge et al., 2009). Munkonge et al. have also reported that the inclusion of NDPK-A and Chx10 binding sequences (BS) adjacent to the SV40 DTS can increase nuclear import of pDNA (Munkonge et al., 2009). Interestingly, additional roles of NDPK-A include activation of *c-myc* transcription and the phosphorylation of GDP to GTP where the phosphate used to enable this reaction is sequestered from an ATP molecule (Dzeja and Terzic, 2003). This is of particular interest in the context of nuclear import, as GTP is required for the migration of cargo through the NPC.

Another major potential bottleneck in TGE derives from the inability of mammalian cells to replicate and maintain extrachromosomal non-viral pDNA throughout culture. This results in dilution of pDNA in a transient setting over time as cells divide in culture and, in turn, can result in limited product yields. The Epstein Barr Virus (EBV) possesses cellular machinery that facilitates replication of its genome in a mammalian host cell (Yates et al., 1985). Successful approaches to enhance TGE yields have focussed on the identification, introduction and optimisation of the necessary EBV derived components to induce extrachromosomal maintenance in transient expression hosts (Yates et al., 1985; Durocher et al., 2002; Backliwal et al., 2008; Daramola et al., 2014). A key component of this system is the Epstein Barr Nuclear Antigen-1 (EBNA-1) protein which, along with a recent discovery of a functional truncated derivative, has been shown to be responsible for partitioning of pDNA with the host chromosome (Durocher et al., 2002). The EBV origin of replication, *oriP*, DNA sequence is required on the expression plasmid for partitioning, but also facilitates the replication of pDNA through “hijacking” of the endogenous machinery that occurs simultaneously to host chromosome replication. Furthermore, two sequences have been identified within the *oriP* region which are involved in EBNA-1 mediated replication. Firstly, a family of repeats (FR) region (consisting of 20 imperfect 30 bp repeats) is the minimal essential region for EBNA-1 partitioning and a dyad symmetry (DS) region (consisting of four 16 bp palindromic repeats contained within a 140 bp sequence) which enhances EBNA-1 binding (Yates et al., 2000). Introduction of the EBNA-1 mediated system has generally proved more successful in human embryonic kidney (HEK) cells (also employed to produce biotherapeutics) than CHO cells, which is not unexpected since EBV natively infects humans but not rodents (Krysan and Calos, 1993; Daramola et al., 2014).

Here we describe two cell engineering approaches to overcome bottlenecks specific to TGE and enhance transient recombinant protein production from CHOK1SV and CHOK1SV GS-KO cells. Firstly, we show that NDPK-A and Chx10 can be overexpressed in CHO cells to improve nuclear import of pDNA and secondly that EBNA-1 mediated extrachromosomal maintenance system can be employed to improve recombinant titres in combination with the nuclear import proteins. Importantly, we show that these engineering approaches, when employed simultaneously, enhance TGE yields beyond the capacity that either technology offers alone when expressing a model IgG4 monoclonal antibody or the SARS-CoV-2 spike (S) protein in CHO cells. Recombinant SARS-CoV-2 S protein is a key reagent used in the production of diagnostics for the virus, against which antibodies are raised for diagnostics, and the main target to date for vaccine development for COVID-19 (Pollet et al., 2021). The TGE technologies outlined herein have thus been employed to increase transient yields of the difficult-to-express trimeric SARS-CoV-2 S protein, highlighting the utility of this platform to rapidly produce such molecules.

## Materials and Methods

### Cloning and Construction of Vectors for Cell Line Engineering and Recombinant Protein Expression

Qiagen’s RNeasy kit was used to isolate RNA from 1 × 10^6^ CHOK1SV cells (Lonza Biologics) and subsequently used to generate cDNA using M-MLV transcriptase (Promega) as per the manufacturers’ instructions. *NDPK-A* and *Chx10* gene sequences were amplified using the polymerase chain reaction (PCR) with the cDNA as the reaction template. The primers designed to amplify *NDPK-A* and *Chx10* were based on NCBI sequence accession numbers XM_027424319.2 and XM_003505140.3, respectively. The primers were also designed to add *Asc*I and *Xho*I restriction sites at the 5′ and 3′ ends of the gene sequences, respectively, to facilitate cloning into proprietary Lonza vectors containing an eGFP reporter or glutamine synthetase (GS) gene (for use as a selection marker). The oligonucleotides used were (restriction site sequences added are underlined); NDPK-A Forward 5′-TATGGCGCGCCATGGCCAACAT-3′ and Reverse 5′-ATACTCGAGTCACTCATAGATCCAGTTTTGTGCACAGCT-3′; Chx10 Forward 5′-TATGGCGCGCCATGACGGGGAA-3′ and Reverse 5′-ATACTCGAGCTAGGCGATGTCCTCCAGCTG-3′. A schematic of the vectors constructed in this study is shown in Supplementary Figure 1.

Complementary binding sequences (BS) sequences for the NLS proteins were also identified (Munkonge et al., 2009) and double stranded DNA fragments for the NDPK-A BS (5′-TATGCGGCCGCGGATCCGGAAGCTTTGGGGAGGGTGG GGAGGGTGGGGAAGGTGGGGAGGAATTCTTTGCAAAA GCCTAGGCCTCCAAGGATCCGCGGCCGCTAT-3′) and Chx10 BS (5′-TATGGATCCGCGGCCGCGGAAGCTTATAACC TAAGCTAATTAGTTATGCATGAATTCTTTGCAAAAGCCTA GGCCTCCAAGCGGCCGCGGATCCTAT-3′) were generated by annealing complementary oligonucleotides. *Bam*HI restriction sites (underlined) were added to these sequences to facilitate cloning into the eGFP-containing vector and previously constructed vectors containing the appropriate NLS containing genes (schematics shown in Supplementary Figure 1).

In order to introduce the EBNA-1 mediated extrachromosomal maintenance system into CHO cells, a truncated *EBNA-1* gene and *oriP* (comprising DS and FR regions) sequences were obtained from vectors generously provided by Prof. Bill Sugden (Reisman et al., 1985; Kirchmaier et al., 1995). The *EBNA-1* gene was amplified via PCR whilst adding *Xba*I and *Age*I restriction sites at the 5′ and 3′ end, respectively, to clone into either the eGFP or GS containing vectors as described above. Individual FR and DS sequences were based on the vectors provided whilst fragments were generated *via* commercial synthesis of the appropriate sequences (GeneART, Invitrogen, United States) where *Bam*HI and *Sal*I restriction sites were added to facilitate cloning. A vector containing the GS (*GLUL*), *EBNA-1* and *NDPK-A* genes was generated to enable cell line engineering of all three components simultaneously.

A gene sequence for expression of a modified version of the SARS-CoV-2 Spike (S) protein was also synthesised commercially (GeneART Invitrogen, United States). This modified version was generated in line with the sequence from Wrapp et al. (2020) where the transmembrane domain has been removed and proline residues introduced to improve stability and remove cleavage sites which are natively sensitive to proteolytic cleavage. The native signal sequence present at the N-terminal of the S protein was substituted for a mammalian signal sequence routinely employed in Lonza’s expression vectors to facilitate efficient secretion and reliable sequence cleavage via signal peptidase in CHO cells. Finally, a His-tag (8 X His) was added to the C-terminus to enable western blot analysis and purification using an anti-His antibody. This modified S protein gene was cloned into the transient vector format described above which contained the *oriP* elements.

### Cell Culture and Cell Line Construction

Lonza CHOK1SV and CHOK1SV GS-KO^®^ host cells were routinely maintained in CD-CHO medium (Thermo Fisher Scientific) supplemented with 6 mM L-glutamine. Transfection of pDNA vectors encoding the gene for GS (*GLUL*) into CHOK1SV or CHOK1SV GS-KO^®^ host cells were achieved by electroporation using a BIORAD GenePulser Xcell electroporator. 1 × 10^7^ viable cells in 700 μL of CD-CHO (Gibco) medium with 20 μg of DNA when only a single vector was transfected (40 μg total linear pDNA was used for co-transfections, 20 μg of each plasmid) was placed in a 0.4 cm BIORAD electroporation cuvette and the exponential protocol was applied using 300 V and 900 μF settings using a BIORAD electroporator. After 24 h recovery in glutamine-free CD-CHO medium, cell pools were selected in glutamine-free CD-CHO medium containing a final concentration of 25 μM methionine sulphoximine (MSX). Cells were sub-cultured every 3–4 days as necessary and seeded at 0.2 × 10^6^ viable cells/mL in a 125 mL Erlenmeyer flask containing a total volume of 20 mL. Cultures were incubated at 37°C with shaking at 140 rpm under a 5% CO_2_ (v/v) gas environment. Cell concentrations and culture viabilities were determined using a ViCell (Beckman Coulter) instrument.

### Flow Cytometry Analysis

Cell samples from suspension cultures analysed by flow cytometry were first centrifuged and resuspended in PBS. Samples were then analysed on a FACScalibur^TM^ instrument (BD Biosciences) and fluorescence intensity measured for 10,000 events. Forward scatter (FSC) was measured using the E-1 amplifier and side scatter (SSC) was set to 465 (Au) whilst FL1 recorded cells at 473 (Au); all settings were converted to a logarithmic scale. Data obtained *via* flow cytometry recorded both the percentage of cells exceeding a predetermined fluorescence threshold to indicate whether cells had been successfully transfected with eGFP expressing vectors and the overall geometric mean fluorescence of all cells recorded. Gating was applied such that Lonza CHOK1SV host cells did not exceed the threshold, but cells transfected with eGFP containing constructs did.

### Western Blot Analysis

Western blot analysis was undertaken essentially as previously described (Roobol et al., 2015). Briefly, SDS-PAGE was used to resolve polypeptides from protein lysates or supernatant samples that were subsequently transferred from the gel to a nitrocellulose membrane. Following this, the membrane was blocked for 30 min in a 5% (w/v) powdered milk solution made up in 0.2% (w/v) Tween TBS. Anti-GFP 3E1 antibody produced in mouse (Research Monoclonal Antibody Service, CRUK), anti-β-actin antibody produced in mouse (Sigma A5441), anti-γ chain antibody produced in rabbit (Sigma I976) and anti-His (Sigma H1029) were the primary antibodies used. Subsequently, anti-Mouse HRP peroxidase (Sigma A4416) and anti-Rabbit HRP peroxidase (Sigma A6154) antibody conjugates were used as secondary detection antibodies. Peroxidase activity on the blot indicated the position of specific immobilised polypeptides on the blot was revealed by chemiluminescence using Hyperfilm ECL reagents (GE Healthcare). Quantitative densitometry was undertaken using ImageJ software.

### Octet Analysis

Supernatants were isolated post transfection from cells that had been transfected with the model IgG4, cB72.3, containing constructs and analysed using an Octet (Fortebio) instrument. Fortebio IgG calibrator standards and protein A biosensors were used to determine IgG concentrations.

### Statistical Analysis

Experiments were undertaken in biological triplicate in all cases by executing three independent transfections for each condition assessed. Error bars represent one standard deviation of the mean. A one way ANOVA and *post hoc* Tukey tests were used to determine statistical significance where appropriate as stated within figure legends.

## Results

### Assessment of Transient Expression of the Genes for *NDPK-A* and *Chx10* and Complementary Consensus Binding Sequences in CHOK1SV Cells

**FIGURE 1:**
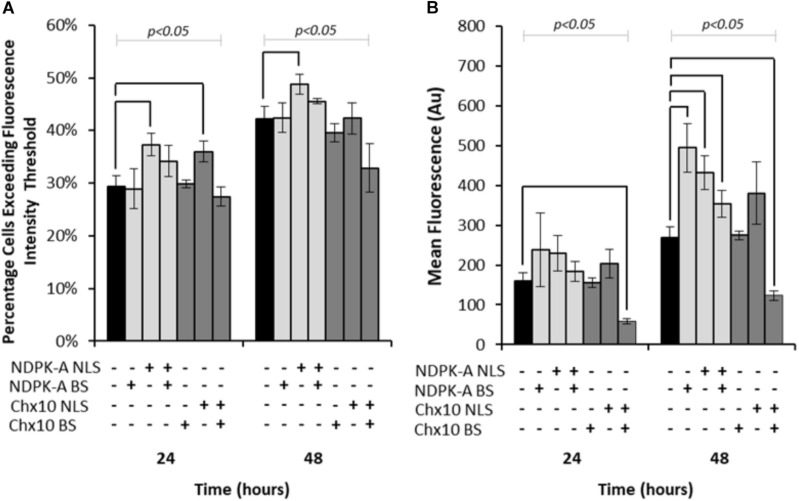
Vectors containing *NDPK-A* and *Chx10* genes that include NLSs, complementary BSs or a combination of the two (vector components present on each vector are indicated through ± below corresponding bars) were transiently electroporated into Lonza CHOK1SV cells and the subsequent fluorescence intensity analysed at 24 and 48 h post transfection via flow cytometry. **(A)** The percentage of cells in the resulting transient populations exceeding a predetermined fluorescence intensity threshold, and **(B)** the overall geometric mean fluorescence. All experiments were run in biological triplicate (*n* = 3) and conditions significantly different from the control condition for each time point are indicated on the graph (*p* < 0.05). A one way ANOVA followed by a *post hoc* Tukey test was used to establish statistical significance to a 95% confidence level. Error bars show ± one standard deviation.

Expression vectors encoding *eGFP* in addition to either *NDPK-A* or *Chx10* genes that contain an NLS, the consensus binding sequence (BS) or a combination of the two were transfected into Lonza’s CHOK1SV cells and analysed by flow cytometry (Figure 1). Both the percentage of cells exceeding a predetermined baseline background fluorescence intensity threshold (from cells not expressing eGFP) and the overall mean fluorescence values were recorded at 24 and 48 h post transfection. The inclusion of the *NDPK-A* gene on the expression vector resulted in an increase in the number of cells exceeding the fluorescence intensity after both 24 and 48 h time points (Figure 1A). Furthermore, there was a higher mean fluorescence from the transient population transfected with the *NDPK-A* NLS containing gene alone when compared to the control after 48 h (Figure 1B). Interestingly, the transfection of NDPK-A construct alone did not significantly increase the percentage of cells exceeding the fluorescence threshold (Figure 1A) but resulted in a 2-fold increase in overall mean fluorescence after 48 h (Figure 1B). A combination of the NDPK-A NLS containing gene construct and BS together resulted in a small increase in the percentage of cells exceeding the fluorescence threshold compared to the control and a statistically significant increase in the overall geometric mean fluorescence value, but this increase was not as substantial as when either component was included alone.

Inclusion of the *Chx10* gene sequence containing an NLS resulted in an increased percentage of cells exceeding the fluorescence intensity threshold after 24 h but this was not the case at the 48 h time point (Figure 1A). Furthermore, this did not translate to a significantly increased geometric mean fluorescence at either time point measured (Figure 1B). The inclusion of the Chx10 BS alone did not yield results that differed from the control but inclusion of both the *Chx10* gene and BS together resulted in generally lower values. Indeed, the geometric mean fluorescence values obtained using this vector and combination were less than half of the control (Figure 1B).

### Inclusion of *EBNA-1* Gene and *oriP* Derived Sequences Results in Attenuated Cell Growth but Increased Fluorescence in Transient CHOK1SV Cultures

**FIGURE 2:**
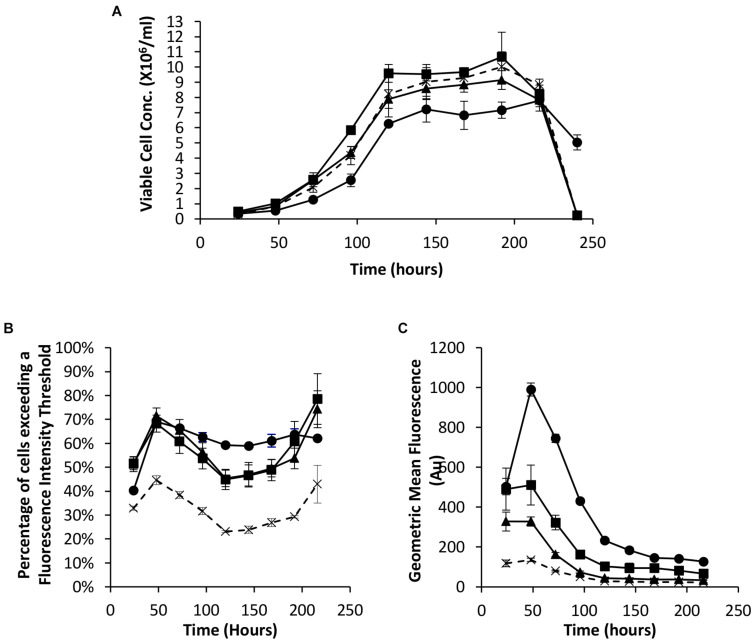
Vectors containing a truncated *EBNA-1* gene, complementary *oriP* elements or a combination of the two were transiently electroporated in Lonza CHOK1SV cells and analysed every 24 hours post transfection for 216 hours. X = control (eGFP only), ◼ = oriP, ▲ = EBNA-1, ● = EBNA-1/oriP. **(A)** Viable cell number of CHOK1SV cells post transfection with the described constructs, **(B)** flow cytometry obtained values showing the percentage of cells of transient populations exceeding a predetermined fluorescence threshold, and **(C)** the overall mean fluorescence, were recorded. Error bars show ± one standard deviation. All experiments were carried out in biological triplicate (*n* = 3).

As described in the introduction, it has been shown that the *EBNA-1* gene can be used to enhance transient expression from CHO cell systems. We therefore established the system here by undertaking transfections with Lonza CHOK1SV cells using pDNA containing either the truncated *EBNA-1* gene, the minimal *oriP* sequence, or a combination of the two on the same plasmid. All vectors also contained the *eGFP* reporter gene to facilitate population analysis using flow cytometry. Figure 2A shows the viable cell numbers recorded over time post transfection. Whilst transfection with constructs bearing *oriP* elements alone resulted in an increase in cell numbers between 72 and 120 h, the inclusion of the *EBNA-1* gene alone resulted in a lower maximum viable cell number on average compared with cells transfected with the control vector. However, transfections carried out with constructs containing both the *EBNA-1* gene and *oriP* sequences resulted in slower growth during exponential phase and lower overall viable cell numbers during stationary phase, but survived longer than cultures transfected with other plasmids.

Samples were also taken at the same time as those used for viable cell number determination in order to evaluate eGFP expression in transient populations by flow cytometry. The inclusion of one or both of the *EBNA-1* gene and *oriP* elements resulted in an increased percentage of cells exceeding the pre-determined fluorescence intensity threshold (Figure 2B) and an increase in the overall geometric mean fluorescence (Figure 2C). Moreover, the inclusion of the vector containing both *EBNA-1* and *oriP* sequences resulted in a 10-fold increase in the geometric mean fluorescence at 48 h post transfection compared to the control and consistently produced the highest geometric mean fluorescent values throughout culture of the combinations investigated in this section.

### Transient Overexpression of NDPK-A With EBNA-1 Mediated Extrachromosomal Maintenance Further Enhances Recombinant Protein Expression

**FIGURE 3:**
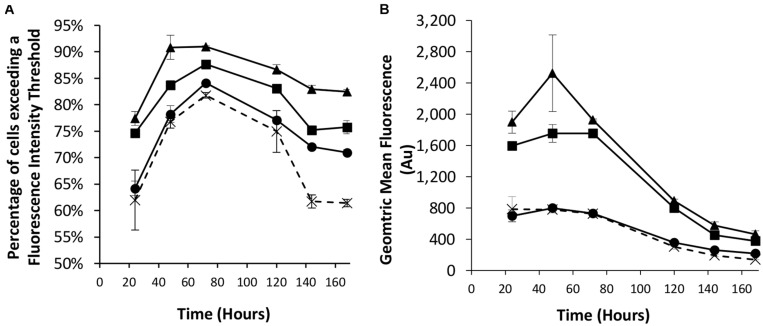
Co-transfections were performed with Lonza CHOK1SV cells using different combinations of control (eGFP alone), NDPK-A NLS and EBNA1/*oriP* vectors. X = control; ● = NDPK-A NLS and control; ◼ = EBNA-1/oriP and control, ▲ = EBNA-1/oriP and NDPK-A NLS (40 μg total DNA was used for each transfection, comprising 20 μg of each specified construct, except in the control samples where 40 μg of the control plasmid was transfected). Flow cytometry was used to assess the transient populations at different time points post transfection to ascertain **(A)** the percentage of cells exceeding a predetermined fluorescence threshold, and **(B)** the overall geometric mean fluorescence. Error bars show ± one standard deviation. All experiments were carried out in biological triplicate (*n* = 3).

We next looked to combine the nuclear shuttling protein expression of NDPK-A with the EBNA-1 based technology into CHOK1SV cells to determine if this could deliver transient expression beyond the capacity of either component individually. As such, different combinations of the eGFP-control, NDPK-A NLS and EBNA-1/*oriP* transient vectors were co-transfected into the Lonza CHOK1SV host cells and the resulting transient populations assessed by flow cytometry. We did not use vectors with the BS on the recombinant (eGFP) vector as the results in section “Assessment of Transient Expression of the Genes for *NDPK-A* and *Chx10* and Complementary Consensus Binding Sequences in CHOK1SV Cells” suggested this did not improve expression over using the *NDPK-A* gene alone. The observed results confirm the data previously described when these approaches were implemented individually (Figures 1, 2). Specifically, the transfection of the *NDPK-A* harbouring vector resulted in a modest increase in the percentage of cells exceeding a fluorescence intensity threshold and inclusion of *EBNA-1* and *oriP* components on the transient vector resulted in a greater increase in both the percentage of cells exceeding the fluorescence intensity threshold and the overall geometric mean fluorescence values (Figures 3A,B). Co-transfection of the *NDPK-A* and *EBNA-1*/*oriP* containing vectors simultaneously resulted in the highest percentage of cells exceeding the fluorescence intensity threshold and overall geometric mean fluorescence. These data show that whilst both technologies can be applied individually to improve transient transfection processes, when these approaches are employed simultaneously this delivers transient expression beyond that observed when either is used individually.

### Stable Engineering of Lonza CHOK1SV GS-KO Cells to Overexpress NDPK-A and Introduce EBNA-1 Mediated Extrachromosomal Maintenance Technologies Enhances Recombinant eGFP Yields

Considering the increased transient expression of GFP when utilising the combined NDPK-A and EBNA-1 mediated extrachromosomal maintenance technologies, Lonza CHOK1SV GS-KO^®^ cells were engineered to either stably overexpress the *NDPK-A* NLS containing gene, express the truncated *EBNA-1* gene, or both. To achieve this, vectors containing these and the GS selection marker gene were used to generate stable pools expressing either NDPK-A alone, truncated EBNA-1 alone, or both as described in “Materials and Methods” section.

**FIGURE 4:**
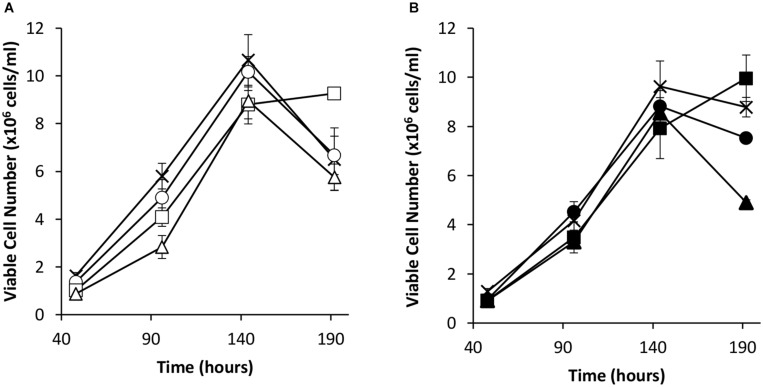
Viable cell number in Lonza CHOK1SV GS-KO cells engineered to overexpress NDPK-A, express EBNA-1 or both (control pools were generated using an empty construct) post-transfection with *eGFP* containing vectors. **(A)** Shows viable cell numbers of transient cultures transfected with a vector containing eGFP alone (X = Control, ○ = NDPK-A, □ = EBNA1 and △ = NDPK-A & EBNA1 cell pools). **(B)** Shows viable cell numbers of transient cultures transfected with a vector containing *eGFP* and *oriP* elements (X = Control, ● = NDPK-A, ◼ = EBNA1 and ▲ = NDPK-A and EBNA1 cell pools). Error bars show ± one standard deviation. All experiments were carried out in biological triplicate (*n* = 3).

Transient transfections were carried out *via* electroporation on these cell pools with a vector bearing the *eGFP* gene alone or the *eGFP* gene and the *oriP* units (FR and DS). The viable cell concentrations of the resulting transient cultures were determined every 48 h for 192 h (Figure 4). As observed in transient experiments (Figure 2A), the inclusion of the *EBNA-1* gene resulted in slowed growth during exponential phase. However, *EBNA-1* expressing pools had the highest viable cell concentrations 192 h post transfection compared to the control in the case of both vectors transiently transfected. Interestingly, EBNA1/NDPK-A pools declined at a faster rate than the control when transfected with the *oriP* containing construct in comparison to other pools (Figure 4B).

**FIGURE 5:**
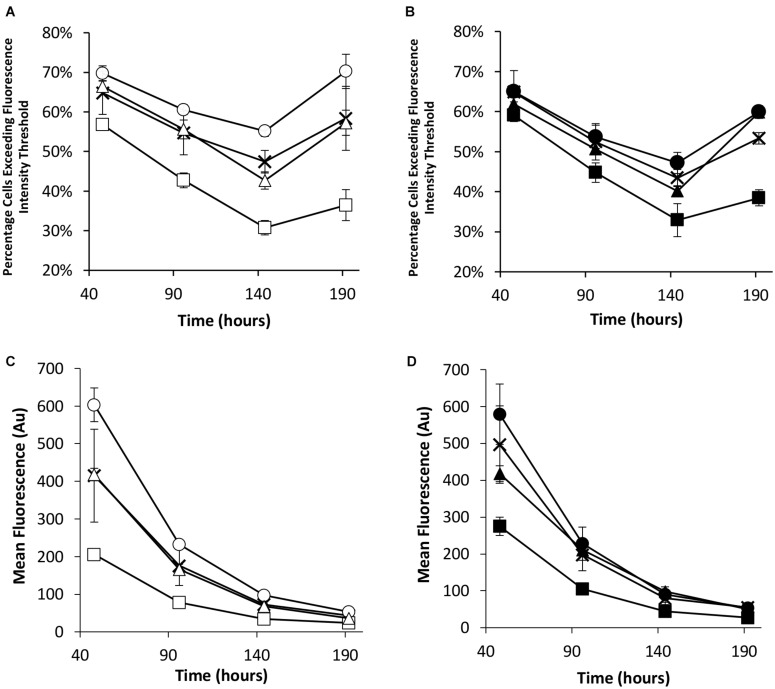
Flow cytometry analysis of Lonza CHOK1SV GS-KO cells engineered to overexpress NDPK-A, express EBNA-1 or both (control pools were generated using an empty construct) post-transfection with eGFP containing vectors. **(A)** Shows the percentage of cells exceeding a fluorescent intensity threshold in transient cultures transfected with a vector containing eGFP alone (X = Control, ○ = NDPK-A, □ = EBNA1 and △ = NDPK-A and EBNA1 cell pools). **(B)** Shows the percentage of cells exceeding a fluorescent intensity threshold in transient cultures transfected with a vector containing eGFP and *oriP* (X = Control, ● = NDPK-A, ◼ = EBNA1 and ▲ = NDPK-A and EBNA1 cell pools). **(C)** Shows the overall geometric mean fluorescence of transient cultures transfected with a vector containing eGFP alone (X = Control, ○ = NDPK-A, □ = EBNA1 and △ = NDPK-A & EBNA1 cell pools). **(D)** Shows the overall geometric mean fluorescence of transient cultures transfected with a vector containing eGFP and *oriP* (X = Control, ● = NDPK-A, ◼ = EBNA1 and ▲ = NDPK-A and EBNA1 cell pools). Error bars show ± one standard deviation. All experiments were carried out in biological triplicate (*n* = 3).

Flow cytometry was used to evaluate the transient eGFP expression at the same time points in the stably engineered CHO cell pools (Figure 5). NDPK-A engineered cell pools yielded the highest percentage of cells exceeding the fluorescence intensity threshold whilst the EBNA-1 cell pools consistently produced the lowest regardless of the presence of *oriP* elements in the transient construct (Figures 5A,B). Furthermore, EBNA-1 expressing pools gave the lowest overall geometric mean fluorescence values across all measured time points, whilst EBNA-1/NDPK-A pools outperformed pools that only expressed EBNA-1 (Figures 5C,D).

**FIGURE 6:**
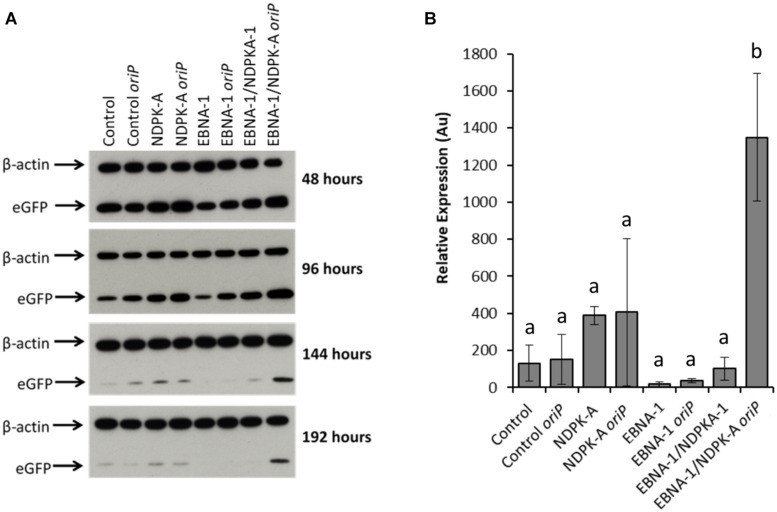
Analysis of Lonza CHOK1SV GS-KO cells engineered to overexpress NDPK-A, express EBNA-1 or both (control pools were generated using an empty construct) post-transfection with eGFP containing vectors. **(A)** Shows western blot analysis of transient cultures transfected with a vector containing eGFP alone or an eGFP*/oriP* vector (denoted by *“oriP*” on the figure). **(B)** Shows relative eGFP abundance from lysates of transient cultures (described above) harvested at 192 h post-transfection. Densitometry analysis was carried out using ImageJ on western blots run in biological triplicate where β-actin was used as a loading control for normalisation. A one way ANOVA test followed by *post hoc* Tukey test was carried out on densitometry data. Conditions which do not share a letter are statistically different at the 95% confidence level. Error bars show ± one standard deviation. All experiments were carried out in biological triplicate (*n* = 3).

Relative eGFP protein levels were also determined using western blot analysis (Figure 6). Lysate samples were obtained at the same time points as those taken for analysis by flow cytometry. The western blot analysis of these samples are shown in Figure 6A, which are a representative of blots analysed in triplicate for all time points. Figure 6B reports the densitometry analysis of western blots of cell lysates harvested at 192 h post-transfection. Overexpression of the *NDPK-A* gene in the Lonza CHOK1SV GS-KO cell pools resulted in enhanced recombinant eGFP yields upon transient transfection with both the eGFP or eGFP/*oriP* vectors that was evident by the increased band intensities observed. This trend was consistent across all time points analysed. Conversely, samples from EBNA-1 only-expressing pools showed less intense eGFP bands compared to the control, although the inclusion of *oriP* elements in the transient vector showed moderate enhancement in these cells. Both these trends were consistent with the previously reported flow cytometry data (Figure 5). A marked increase in relative eGFP yields was evident across all time points when EBNA1/NDPK-A expressing cell pools were transiently transfected with the *oriP*-containing vector. Not only did these cells show an initial increase in relative eGFP levels at 48 h post transfection, an approximate 10-fold increase in expression levels was still evident at 192 h post-transfection (Figure 6B). Furthermore, the comparatively low levels of eGFP observed upon transfection of the vector comprising eGFP alone in EBNA1/NDPK-A expressing cell pools highlights the requirements of the *oriP* components in the transient vector.

### Stable Expression of NDPK-A and EBNA-1 in CHO Cells and Inclusion of *oriP* Elements on Transient Vectors Enhances Production of a Model IgG4 Molecule and the SARS-CoV-2 Spike Protein

**FIGURE 7:**
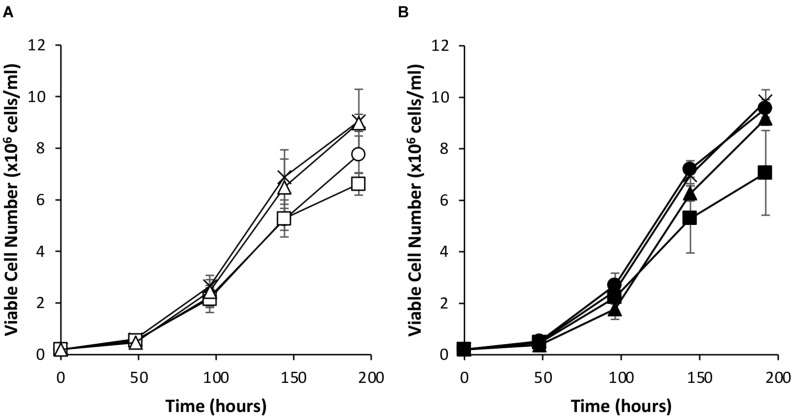
Viable cell number of Lonza CHOK1SV GS-KO cells engineered to overexpress NDPK-A, express EBNA-1 or both (control pools were generated using an empty construct) post-transfection with vectors containing genes for a model IgG4 mAb. **(A)** Shows viable cell numbers of transient cultures transfected with a vector containing the mAb genes alone (X = Control, ○ = NDPK-A, □ = EBNA1 and △ = NDPK-A and EBNA1 cell pools). **(B)** Shows viable cell numbers of transient cultures transfected with a vector containing mAb genes and *oriP* elements (X = Control, ● = NDPK-A, ◼ = EBNA1 and ▲ = NDPK-A and EBNA1 cell pools). Error bars show ± one standard deviation. All experiments were carried out in biological triplicate (*n* = 3).

**FIGURE 8:**
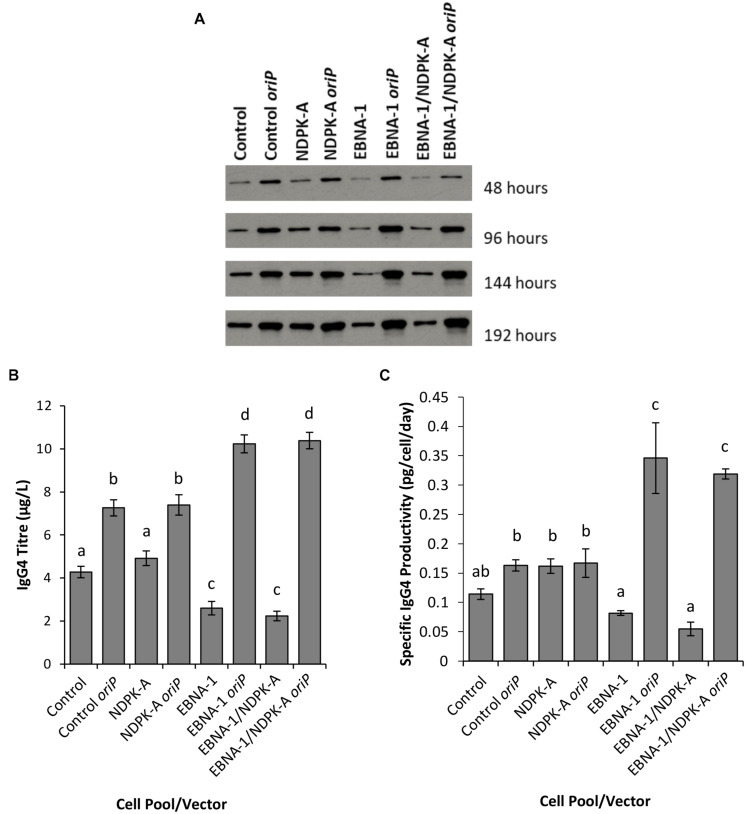
Analysis of Lonza CHOK1SV GS-KO cells engineered to overexpress NDPK-A, express EBNA-1 or both (control pools were generated using an empty construct) post-transfection with vectors containing genes for a model IgG mAb. **(A)** Shows western blot analysis of transient cultures transfected with a vector containing mAb genes alone or a mAb*/oriP* vector (denoted by *“oriP*” on the figure). **(B)** Shows relative eGFP abundance from lysates of transient cultures (described above) harvested at 192 hours post-transfection. Quantification of IgG mAb was carried out using Octet analysis. A one way ANOVA test followed by *post hoc* Tukey test was carried out on titre data **(B,C)**. Conditions which do not share a letter are statistically different at the 95% confidence level. Error bars show ± one standard deviation. All experiments were carried out in biological triplicate (*n* = 3).

Since the simultaneous introduction of EBNA-1 and NDPK-A into CHOK1SV GS-KO cells proved effective in enhancing expression of an eGFP reporter, the system was evaluated using an industrially relevant model monoclonal antibody, an IgG4 molecule (cB72.3). Transient transfections were carried out using vectors that contained the genes necessary for expression of the IgG where the *oriP* elements were either included or excluded (Figures 7, 8). Viable cell concentrations were recorded every 48 h post transfection (Figure 7). Cell pools engineered to express EBNA-1 alone showed lower viable cell numbers when compared to the control host pools regardless of whether the transient vector included the *oriP* elements or not. This observation is consistent with the previously described data where engineered cell pools were transfected with eGFP containing vectors (Figure 4).

Supernatants from batch culture were harvested every 48 h for 192 h post transfection and assessed using western blot (Figure 8A) and Octet Protein A analysis in order to determine quantity of IgG4 secreted into culture medium (Figure 8B) and to calculate pool productivity (Figure 8C). The inclusion of *oriP* elements on the expression vector resulted in a general increase in the concentration of IgG4 observed, and this was the case for all cell pools analysed. However, the inclusion of the *oriP* elements resulted in a far greater increase in IgG4 volumetric product concentration than the control vector in both EBNA1 and EBNA-1/NDPK-A cell pools; approximately an 8-fold increase in product titre was observed in these pools in comparison to <2-fold increase observed in the control cell pools (Figure 8B). Moreover, transfections with the IgG4-only (no *oriP*) vector resulted in a significantly lower titre in EBNA1 and EBNA1/NDPK-A cell pools compared to the control.

The NDPK-A overexpressing cell pool showed higher specific productivity than the control when transfected with the IgG4-only vector (Figure 8C) but this did not result in improved volumetric productivity (Figure 8B) due to the lower viable cell concentrations achieved (Figure 7A). Furthermore, the overexpression of NDPK-A in combination with the introduction of EBNA1 did not result in significantly greater IgG4 product yields than what was achieved in the pool engineered with EBNA1 alone (Figure 8B) as previously observed with the eGFP containing constructs (Figure 6).

We then applied the system to the expression of the SARS-CoV-2 Spike or S protein which is a large glycosylated protein that is difficult to express (Johari et al., 2021). Viral entry is mediated by binding of the spike (S) protein that protrudes from the surface of the pathogen and binds to angiotensin-converting enzyme 2 (ACE2) present on the surface of host cells (Lan et al., 2020). The S protein is a key target for both vaccine design and diagnostics due its central role in facilitating infection and its presence on the outer membrane of the virus means it is accessible to the immune system. The S protein is a multi-domain and heavily glycosylated protein and production of material with similar attributes is crucial for effective application of the S protein as a part of a vaccine or diagnostics. CHO cells are capable of generating such material with suitable properties (Johari et al., 2021). Therefore, following successful implementation of NDPK-A and EBNA-1 based technologies in CHO cells to generate enhanced yields of reporter and model mAb proteins, we employed this platform to generate the SARS-CoV-2 S protein that had been engineered to remove the native transmembrane domain and proteolytic cleavage sites (Wrapp et al., 2020).

**FIGURE 9:**
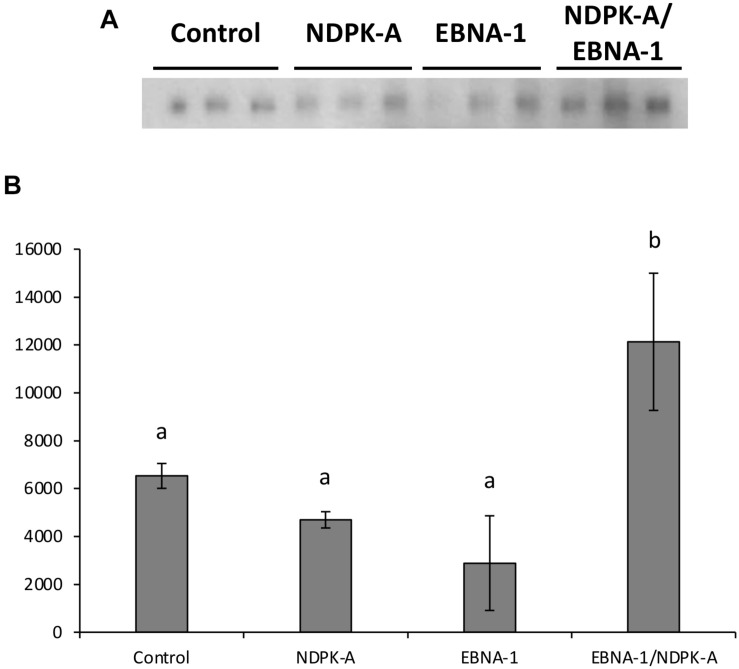
Analysis of Lonza CHOK1SV GS-KO cells engineered to overexpress NDPK-A, express EBNA-1 or both (control pools were generated using an empty construct) post-transfection with vectors containing the gene for a SARS nCov Spike and the *oriP* elements. **(A)** Shows western blot analysis of supernatants harvested 8 days post-transfection using an anti-His tag antibody, whilst figure **(B)** shows relative band intensities as determined using densitometry. A one way ANOVA test followed by *post hoc* Tukey test was carried out on densitometry data. Conditions which do not share a letter are statistically different at the 95% confidence level. Error bars show ± one standard deviation. All experiments were carried out in biological triplicate (*n* = 3).

Figure 9A shows western blot analysis of supernatants, harvested 8 days post-transfection, of the engineered CHO cell pools (control, NDPK-A, EBNA-1, or EBNA-1/NDPK-A) grown under batch culture conditions and transiently transfected with an expression vector containing the modified SARS-CoV-2 S protein and minimal oriP elements (FR and DS). The bands observed were revealed by probing the blot with an anti-His antibody (Sigma H1029) and densitometry carried out on bands to determine the relative quantities of modified S protein generated using the different cell pools. The cell pools which were engineered to overexpress either NDPK-A or introduce EBNA-1 independently produced less modified S protein than the control which was not engineered with either gene. However, the cell pool which was engineered to overexpress NDPK-A and EBNA-1 simultaneously resulted in a 1.8-fold increase in Spike protein yields on average than the non-engineered control cell pool (Figure 9B).

## Discussion

The advantages of using TGE to rapidly and cost effectively produce material for pre-clinical studies has long been recognised, but development of transient expression platforms capable of producing high yields of efficacious material has gained increasing focus in recent years in light of the increased number of products entering clinical trials. Furthermore, it is beneficial to employ the same host cell line for both transient and stable production so that the material generated for drug candidate evaluation in transient experiments is representative of the final stably produced product. Indeed, recent advances in CHO cell TGE technologies and associated enhanced product yields has led to questions around whether such transient platforms could be used to generate specific materials for clinical phase 1 trials or in rapid response to pandemics (Gutiérrez-Granados et al., 2018). TGE is typically used for smaller scale production where the speed of production is key. There are relatively fewer reports of large scale transient production due to the high cost of the process in comparison to the protein yield obtained (Baldi et al., 2007). By improving transient yields, the cost efficiency of the process is increased but the overall yields obtained are not yet comparable to those generated using stable production technologies, although for “easier” to express standard monoclonal antibodies yields in the g/L have been reported.

A key aspect of transient systems is the delivery of DNA into the nucleus and then the subsequent maintenance of this within the nucleus. Although there have now been a number of studies focussed on improving nuclear maintenance, there have been few, if any advances in improving DNA entry into the nucleus. Indeed, import of DNA across the nuclear barrier is poorly understood, although it has been hypothesised that this process could be either passive, i.e., when the nuclear membrane is compromised during cell division, or active through the NPC. Whilst the exact weight of contribution of these two mechanisms has not been determined, nuclear import of pDNA has been shown to be maximal in actively proliferating cells (Edwards and Passineau, 2015). Moreover, other factors may contribute to the mechanism or efficiency of nuclear entry, e.g., size of pDNA and whether the pDNA is delivered as part of a polyplex (as for example, in chemically-based transfection methods) or transfected as naked DNA (in physically based transfection methods such as electroporation). Nonetheless, it is thought that entry of pDNA into the nucleus is a potential limitation in transient expression. Here, we have described a method for improved nuclear import through the NPC by overexpressing either NDPK-A or Chx10 transcription factors in CHO cells. These proteins have previously been shown to facilitate nuclear import in non-dividing HeLa cells (Munkonge et al., 2009). The inclusion of a consensus BS on the transient plasmid did not necessarily improve the CHO cell transient system. In fact, when the Chx10 NLS and BS were both included on the transient vector there was a significant decrease in both the percentage of cells above a fluorescence intensity threshold and mean fluorescence intensity. The NDPK-A and Chx10 BSs were previously shown to improve nuclear import when included on pDNA adjacent to the SV40 DTS. In this study, the Chx10 BS did not function as a traditional DTS when positioned away from an existing DTS on the pDNA suggesting that this consensus sequence cannot be used as a DTS independently. The inclusion of the NDPK-A NLS and BS simultaneously did not show any advantage over including either independently.

The inclusion of the *NDPK-A* gene alone (without BS) improved both the percentage and overall fluorescence intensity following transient transfection with an eGFP containing plasmid when transiently and stably employed. In addition to its function in nuclear import, NDPK-A also plays a major role in the transfer of a phosphate group from ATP to a nucleoside diphosphate other than ATP; such as the synthesis of GTP from GDP (Boissan et al., 2014). In a nuclear import context, GTP is required to supply energy for the migration of cytoplasmic cargo through the nuclear pore complex into the nucleus (Dzeja and Terzic, 2003). Overexpression of the NDPK-A gene may therefore upregulate the rate at which molecules can traverse the nuclear membrane due to improved availability of energy from GTP. NDPK-A also impacts cell proliferation and is functional as a transcription factor (Sharma et al., 2018) and thus, although differences observed in reporter protein expression levels are likely due to increased transfection efficiencies and delivery of pDNA into the nucleus, it is possible that the increase observed may be owed, at least in part, to these other roles of NDPK-A.

Introduction of the Epstein Barr derived EBNA-1 protein and the essential *oriP* (FR and DS) sequences have previously proved successful in inducing extrachromosomal maintenance in mammalian cells (Längle-Rouault et al., 1998; Van Craenenbroeck et al., 2000; Yates et al., 2000; Backliwal et al., 2008; Jäger et al., 2013; Daramola et al., 2014) although this approach has generally been more successful in human cells such as human embryonic kidney (HEK) cells than in CHO cells (Kunaparaju et al., 2005). However, as described above, reports have suggested that the rate of nuclear import of plasmid DNA is a potential bottleneck in hosts engineered to introduce the EBNA-1 driven extrachromosomal system (Carpentier et al., 2007). Our data support this, but further, also suggests that a decrease in the rate of nuclear import of plasmid DNA may be linked to the reduced growth observed when the EBNA-1 gene is introduced in the Lonza CHOK1SV or CHOK1SV GS-KO cells either transiently or stably, respectively. Reduced growth, and hence proliferation, will result in less frequent breakdown of the nuclear envelope during M phase of the cell cycle and therefore a lower passive rate of nuclear entry of plasmid DNA during transfection. Furthermore, the introduction of the EBNA-1 extrachromosomal maintenance system may place additional metabolic burden upon the CHO cell host and could account for observed reduced growth. The data presented here shows that cells only expressing exogenously added EBNA-1 gene have compromised growth and consistently decreased expression of both eGFP and an IgG4 molecules in the absence of the *oriP* elements.

NDPK-A and EBNA-1 based technologies were introduced simultaneously following their successful application independently. Both transient and stable employment of these approaches proved effective in improving eGFP yields. Following successful transient transfection of CHOK1SV cells with expression vectors encoding eGFP, EBNA-1, NDPK-A and *oriP*, transient transfection of CHOK1SV GS-KO pools co-expressing EBNA-1 and NDPK-A with expression vectors encoding *oriP* and eGFP, led to a 10-fold increase in eGFP abundance compared to the control. Whilst the NDPK-A/EBNA-1 system was able to improve transient yields of the IgG4 molecule compared to the non-engineered control, no improvement was observed beyond that observed when the EBNA-1 system was implemented alone. When employed independently, the NDPK-A driven technology was able to modestly enhance IgG4 productivity compared to the control. Collectively these data suggest it is likely that transient expression yields are determined by different bottlenecks dependant on the molecule being expressed and transfection efficiency may be at least partially, impacted by the size of the vector as previously reported (Kreiss et al., 1999; Hornstein et al., 2016). However, when we used the system to express another recombinant protein from a construct close in size to the IgG4 construct, that for the S protein (Supplementary Figure 1), we did see an increase in transient expression suggesting size is not a major constraint. Indeed, when the EBNA-1 and NDPK-A mediated technologies were employed to generate a modified version of the SARS-CoV-2 S protein the cells engineered with the technologies separately (i.e., EBNA-1-CHO and NDPK-A-CHO cells) generated less S protein than the control. However, the cells engineered to contain both technologies (EBNA-1/NDPK-A-CHO) gave a 1.8-fold increase in the expression of secreted S-protein compared to the control. This provides further evidence that these technologies can be implemented simultaneously to achieve higher secretory recombinant protein yields of molecules of various different formats whilst the same technologies implemented independently did not always generate more material. The recombinant production of S protein is of particular interest since it is a key component in manufacturing diagnostics for SARS-CoV-2 and a key target for development of COVID-19 vaccines (Pollet et al., 2021). By employing the TGE system outlined here, more S protein was produced than the original CHO host transient system, advantageous for rapid generation of material for diagnostics, antibody development and for evaluation of potential vaccine candidates. This is therefore an example of how this TGE platform could be utilised to accelerate vaccine and biotherapeutic discovery timelines.

## Conclusion

In conclusion, we have shown that EBNA-1 and NDPK-A based technologies can be employed independently or simultaneously to enhance transient yields from CHO cells, and particularly of the difficult to express SARS-CoV-2 S protein. The increase in secretory productivity from these technologies is dependent on the molecule of interested being transiently expressed which is not surprising given different molecules are known to present different cellular challenges and bottlenecks for optimal yields. Nevertheless, this study shows that a combined approach of nuclear DNA maintenance coupled with enhanced plasmid DNA nuclear entry is a promising concept to further enhance the transient yields of recombinant protein that can be achieved from CHO cells and can be used to obtain higher yields of complex glycosylated proteins such as the S protein.

## Data Availability Statement

The raw data supporting the conclusions of this article will be made available by the authors, without undue reservation.

## Author Contributions

JB was involved in the planning of experiments, executed experiments, interpreted data, and drafted the manuscript and figures. RY and CS conceived the initial study and both planned experiments and interpreted data with JB. CS helped draft the manuscript with JB. All authors proofed the manuscript, data, and figures and approved its submission.

## Conflict of Interest

RY, at the time of this work, was employed by Lonza Biologics, who developed and license the GS Gene Expression System. Lonza is the assigned owner of, and CS, JB, and RJ are named inventors on, the filed patent application “Mammalian expression system,” patent number WO2017032834A1.

## Supplementary Material

Supplementary Figure 1 Details of vectors generated and used for transient studies including the vector name, schematic of components of the construct, and vector size in base pairs (bp).

## References

[B1] Backliwal G. Hildinger M. Chenuet S. Wulhfard S. De Jesus M. Wurm F. M. (2008). Rational vector design and multi-pathway modulation of HEK 293E cells yield recombinant antibody titers exceeding 1 g/l by transient transfection under serum-free conditions. *Nucleic Acids Res.* 36:e96. 10.1093/nar/gkn423 18617574 PMC2528171

[B2] Baldi L. Hacker D. L. Adam M. Wurm F. M. (2007). Recombinant protein production by large-scale transient gene expression in mammalian cells: state of the art and future perspectives. *Biotechnol. Lett.* 29 677–684. 10.1007/s10529-006-9297-y 17235486

[B3] Boissan M. Montagnac G. Shen Q. Griparic L. Guitton J. Romao M. (2014). Membrane trafficking. Nucleoside diphosphate kinases fuel dynamin superfamily proteins with GTP for membrane remodeling. *Science* 344 1510–1515. 10.1126/science.1253768 24970086 PMC4601533

[B4] Budge J. D. Knight T. J. Povey J. Roobol J. Brown I. R. Singh G. (2019). Engineering of Chinese hamster ovary cell lipid metabolism results in an expanded ER and enhanced recombinant biotherapeutic protein production. *Metab. Eng.* 57 203–216. 10.1016/j.ymben.2019.11.007 31805379 PMC6975165

[B5] Carpentier E. Paris S. Kamen A. A. Durocher Y. (2007). Limiting factors governing protein expression following polyethylenimine-mediated gene transfer in HEK293-EBNA1 cells. *J. Biotechnol.* 128 268–280. 10.1016/J.JBIOTEC.2006.10.014 17118475

[B6] Daramola O. Stevenson J. Dean G. Hatton D. Pettman G. Holmes W. (2014). A high-yielding CHO transient system: coexpression of genes encoding EBNA-1 and GS enhances transient protein expression. *Biotechnol. Prog.* 30 132–141. 10.1002/btpr.1809 24106171

[B7] Dean D. A. (1997). Import of plasmid DNA into the nucleus is sequence specific. *Exp. Cell Res.* 230 293–302. 10.1006/excr.1996.3427 9024788

[B8] Dean D. A. Strong D. D. Zimmer W. E. (2005). Nuclear entry of nonviral vectors. *Gene Ther.* 12 881–890. 10.1038/sj.gt.3302534 15908994 PMC4403635

[B9] Durocher Y. Perret S. Kamen A. (2002). High-level and high-throughput recombinant protein production by transient transfection of suspension-growing human 293-EBNA1 cells. *Nucleic Acids Res.* 30:E9. 10.1093/nar/30.2.e9 PMC99848 11788735

[B10] Dzeja P. P. Terzic A. (2003). Phosphotransfer networks and cellular energetics. *J. Exp. Biol.* 206 2039–2047. 10.1242/jeb.00426 12756286

[B11] Edwards P. C. Passineau M. J. (2015). *Genetic Manipulation Via Gene Transfer, in Stem Cell Biology and Tissue Engineering in Dental Sciences.* Amsterdam: Elsevier Inc, 75–84. 10.1016/B978-0-12-397157-9.00008-4

[B12] Gasiorowski J. Z. Dean D. A. (2003). Mechanisms of nuclear transport and interventions. *Adv. Drug Deliv. Rev.* 55 703–716. 12788535 10.1016/s0169-409x(03)00048-6

[B13] Godfrey C. L. Mead E. J. Daramola O. Dunn S. Hatton D. Field R. (2017). Polysome profiling of mAb producing CHO cell lines links translational control of cell proliferation and recombinant mRNA loading onto ribosomes with global and recombinant protein synthesis. *Biotechnol. J.* 12:1700177. 10.1002/biot.201700177 28504349

[B14] Gourbatsi E. Al-Fageeh M. B. Marchant R. J. Scott S. J. Underhill M. F. Smales C. M. (2006). Noncovalently linked nuclear localization peptides for enhanced calcium phosphate transfection. *Mol. Biotechnol.* 33 1–11. 10.1385/MB:33:1:1 16691001

[B15] Gutiérrez-Granados S. Cervera L. Kamen A. A. Gòdia F. (2018). Advancements in mammalian cell transient gene expression (TGE) technology for accelerated production of biologics. *Crit. Rev. Biotechnol.* 38 918–940. 10.1080/07388551.2017.1419459 29295632

[B16] Hornstein B. D. Roman D. Arévalo-Soliz L. M. Engevik M. A. Zechiedrich L. (2016). Effects of circular DNA length on transfection efficiency by electroporation into HeLa cells. *PLoS One* 11:e0167537. 10.1371/journal.pone.0167537 27918590 PMC5137892

[B17] Jäger V. Büssow K. Wagner A. Weber S. Hust M. Frenzel A. (2013). High level transient production of recombinant antibodies and antibody fusion proteins in HEK293 cells. *BMC Biotechnol.* 13:52. 10.1186/1472-6750-13-52 23802841 PMC3699382

[B18] Johari Y. B. Jaffé S. R. P. Scarrott J. M. Johnson A. O. Mozzanino T. Pohle T. H. (2021). Production of trimeric SARS-CoV-2 spike protein by CHO cells for serological COVID-19 testing. *Biotechnol. Bioeng.* 118 1013–1021. 10.1002/bit.27615 33128388

[B19] Kirchmaier A. L. Loss T. A. B. Mackey D. Sugden B. (1995). Plasmid maintenance of derivatives of *oriP* of Epstein-Barr virus. *J. Virol.* 69 1280–1283. 10.1128/JVI.69.2.1280-1283.1995 7815506 PMC188704

[B20] Kreiss P. Mailhe P. Scherman D. Pitard B. Cameron B. Rangara R. (1999). Plasmid DNA size does not affect the physicochemical properties of lipoplexes but modulates gene transfer efficiency. *Nucleic Acids Res.* 27 3792–3798. 10.1093/nar/27.19.3792 10481017 PMC148641

[B21] Krysan P. J. Calos M. P. (1993). Epstein-Barr virus-based vectors that replicate in rodent cells. *Gene* 136 137–143. 10.1016/0378-1119(93)90457-e 8293997

[B22] Kunaparaju R. Liao M. Sunstrom N. (2005). *Epi* -CHO, an episomal expression system for recombinant protein production in CHO cells. *Biotechnol. Bioeng.* 91 670–677. 10.1002/bit.20534 15948170

[B23] Lan J. Ge J. Yu J. Shan S. Zhou H. Fan S. (2020). Structure of the SARS-CoV-2 spike receptor-binding domain bound to the ACE2 receptor. *Nature* 581 215–220. 10.1038/s41586-020-2180-5 32225176

[B24] Längle-Rouault F. Patzel V. Benavente A. Taillez M. Silvestre N. Bompard A. (1998). Up to 100-fold increase of apparent gene expression in the presence of Epstein-Barr virus oriP sequences and EBNA1: implications of the nuclear import of plasmids. *J. Virol.* 72 6181–6185. 10.1128/JVI.72.7.6181-6185.1998 9621086 PMC110430

[B25] Marichal-Gallardo P. A. Álvarez M. M. (2012). State-of-the-art in downstream processing of monoclonal antibodies: process trends in design and validation. *Biotechnol. Prog.* 28 899–916. 10.1002/btpr.1567 22641473

[B26] Munkonge F. M. Amin V. Hyde S. C. Green A.-M. Pringle I. A. Gill D. R. (2009). Identification and functional characterization of cytoplasmic determinants of plasmid DNA nuclear import. *J. Biol. Chem.* 284 26978–26987. 10.1074/jbc.M109.034850 19638341 PMC2785383

[B27] Pemberton L. F. Paschal B. M. (2005). Mechanisms of receptor-mediated nuclear import and nuclear export. *Traffic* 6 187–198. 10.1111/j.1600-0854.2005.00270.x 15702987

[B28] Pollard H. Remy J. S. Loussouarn G. Demolombe S. Behr J. P. Escande D. (1998). Polyethylenimine but not cationic lipids promotes transgene delivery to the nucleus in mammalian cells. *J. Biol. Chem.* 273 7507–7511. 10.1074/jbc.273.13.7507 9516451

[B29] Pollet J. Chen W. H. Strych U. (2021). Recombinant protein vaccines, a proven approach against coronavirus pandemics. *Adv. Drug Deliv. Rev.* 170 71–82. 10.1016/j.addr.2021.01.001 33421475 PMC7788321

[B30] Povey J. F. O’Malley C. J. Root T. Martin E. B. Montague G. A. Feary M. (2014). Rapid high-throughput characterisation, classification and selection of recombinant mammalian cell line phenotypes using intact cell MALDI-ToF mass spectrometry fingerprinting and PLS-DA modelling. *J. Biotechnol.* 184 84–93. 10.1016/j.jbiotec.2014.04.028 24858576

[B31] Reisman D. Yates J. Sugden B. (1985). A putative origin of replication of plasmids derived from Epstein-Barr virus is composed of two cis-acting components. *Mol. Cell. Biol.* 5 1822–1832. 10.1128/mcb.5.8.1822 3018528 PMC366897

[B32] Roobol A. Roobol J. Bastide A. Knight J. R. P. Willis A. E. Smales C. M. (2015). p58IPK is an inhibitor of the eIF2α kinase GCN2 and its localization and expression underpin protein synthesis and ER processing capacity. *Biochem. J.* 465 213–225. 10.1042/BJ20140852 25329545

[B33] Sharma S. Sengupta A. Chowdhury S. (2018). NM23/NDPK proteins in transcription regulatory functions and chromatin modulation: emerging trends. *Lab. Invest.* 98 175–181. 10.1038/labinvest.2017.98 29083410 PMC5854247

[B34] Subramanian A. Ranganathan P. Diamond S. L. (1999). Nuclear targeting peptide scaffolds for lipofection of nondividing mammalian cells. *Nat. Biotechnol.* 17 873–877. 10.1038/12860 10471928

[B35] Van Craenenbroeck K. Vanhoenacker P. Haegeman G. (2000). Episomal vectors for gene expression in mammalian cells. *Eur. J. Biochem.* 267 5665–5678. 10.1046/j.1432-1327.2000.01645.x 10971576

[B36] Walsh G. (2018). Biopharmaceutical benchmarks 2018. *Nat. Biotechnol.* 36 1136–1145. 10.1038/nbt.4305 30520869

[B37] Wrapp D. Wang N. Corbett K. S. Goldsmith J. A. Hsieh C.-L. Abiona O. (2020). Cryo-EM structure of the 2019-nCoV Spike in the prefusion conformation. *Science* 367 1260–1263. 10.1126/science.abb2507 32075877 PMC7164637

[B38] Wurm F. M. (2004). Production of recombinant protein therapeutics in cultivated mammalian cells. *Nat. Biotechnol.* 22 1393–1398. 10.1038/nbt1026 15529164

[B39] Yates J. L. Camiolo S. M. Bashaw J. M. (2000). The minimal replicator of Epstein-Barr virus oriP. *J. Virol.* 74 4512–4522. 10.1128/jvi.74.10.4512-4522.2000 10775587 PMC111971

[B40] Yates J. L. Warren N. Sugden B. (1985). Stable replication of plasmids derived from Epstein–Barr virus in various mammalian cells. *Nature* 313 812–815. 10.1038/313812a0 2983224

[B41] Zanta M. A. Belguise-Valladier P. Behr J. P. (1999). Gene delivery: a single nuclear localization signal peptide is sufficient to carry DNA to the cell nucleus. *Proc. Natl. Acad. Sci. U. S. A.* 96 91–96. 10.1073/pnas.96.1.91 9874777 PMC15098

