# Supplementary figures and images for: Engineering of Chinese Hamster Ovary Cells With NDPK-A to Enhance DNA Nuclear Delivery Combined With EBNA1 Plasmid Maintenance Gives Improved Exogenous Transient Reporter, mAb and SARS-CoV-2 Spike Protein Expression
Source: Front Bioeng Biotechnol. 2021 Jun 4;9:679448. doi: 10.3389/fbioe.2021.679448 (PMC8212061; doi:10.3389/fbioe.2021.679448)

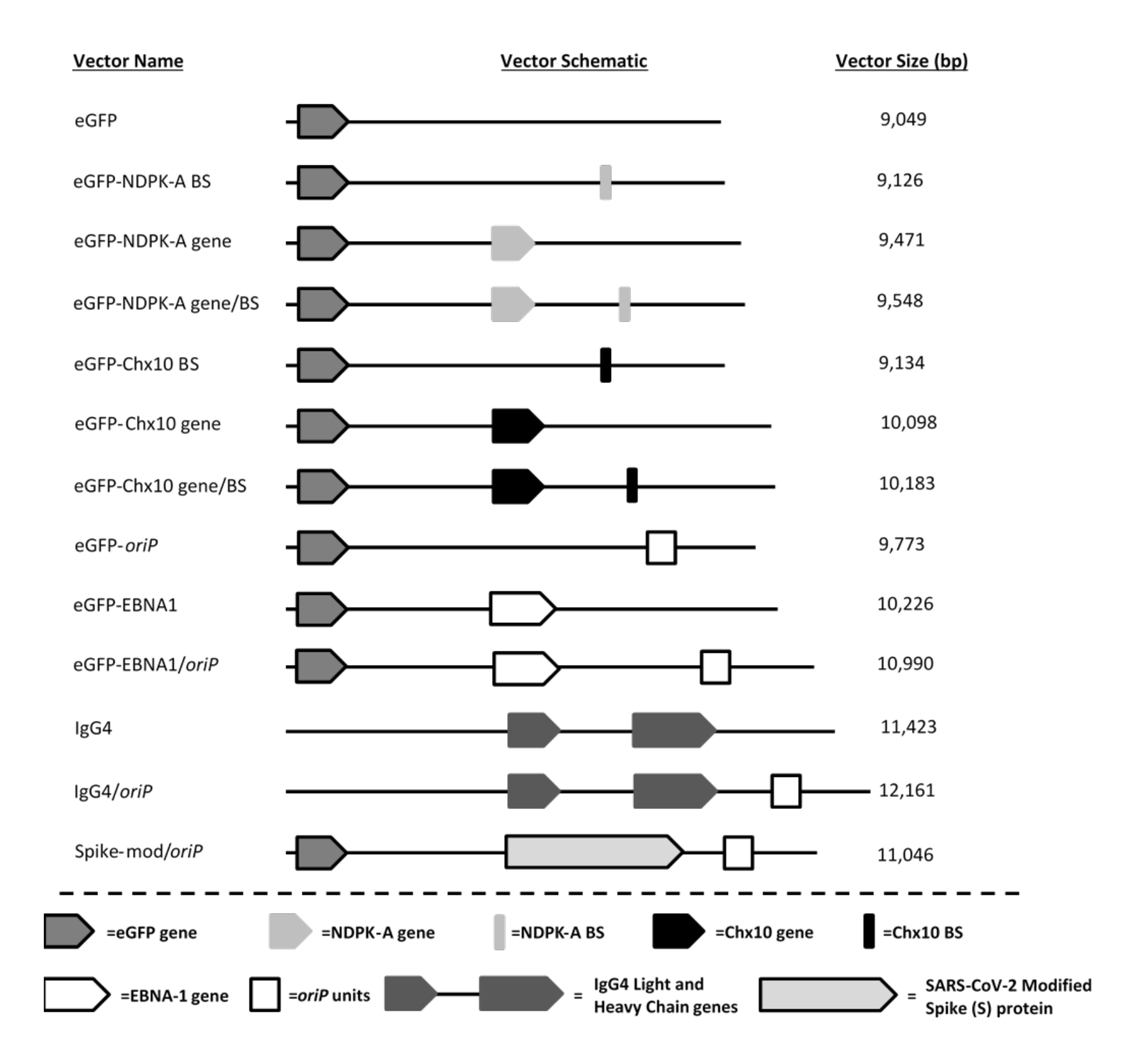

Supplement: Supplementary Figure 1 — Details of vectors generated and used for transient studies including the vector name, schematic of components of the construct, and vector size in base pairs (bp). [file Image_1.TIF]
